# Zinc Finger Protein 143 Bidirectionally Regulates the Proliferation of Hepatocytes and Hepatocellular Carcinoma Cells via c‐Myc

**DOI:** 10.1002/cbf.70320

**Published:** 2026-10-07

**Authors:** Caifang Sun, Meijuan Yue, Yuxiang Chen, Jiaying Zhang, Yunyun Mai, Yuhang Zhang, Bingyu Ye

**Affiliations:** ^1^ State Key Laboratory of Cell Differentiation and Regulation, College of Life Sciences Henan Normal University Xinxiang China

**Keywords:** cell cycle, cell proliferation, c‐Myc, liver regeneration, ZNF143

## Abstract

The transcription factor zinc finger protein 143 (ZNF143) plays a crucial role in regulating diverse biological processes, including cell cycle progression, development, and cellular differentiation. Similarly, c‐Myc is well‐established as a key regulator of cell proliferation, differentiation, and metabolic homeostasis. While studies have demonstrated ZNF143's involvement in the proliferation of both normal hepatocytes and hepatoma cells, the precise mechanisms underlying ZNF143‐mediated regulation of hepatocyte proliferation, as well as its potential functional interplay with c‐Myc in this process, remain to be fully elucidated. In this study, we investigated the dynamic expression patterns of ZNF143 and c‐Myc during distinct phases of liver regeneration using a 2/3 partial hepatectomy (PHx) mouse model. To elucidate the regulatory roles of these factors in hepatocyte proliferation, we employed the mouse normal hepatocyte line AML12 and the human hepatoma cell line Huh7 as experimental models. Through MTT, CCK‐8, EdU, quantitative real‐time PCR (qRT‐PCR), and Western blot, we examined the individual and combined effects of ZNF143 and c‐Myc on hepatocyte proliferation. Our findings reveal that ZNF143 inhibits proliferation and cell cycle progression in AML12 cells but promotes these processes in Huh7 cells. Notably, co‐overexpression (OE) of ZNF143 and c‐Myc in AML12 cells inhibited proliferation and cell cycle progression, suggesting that ZNF143 exerts a dominant regulatory effect over c‐Myc in normal hepatocytes. This study establishes a critical foundation for investigating the role of ZNF143 in hepatocyte proliferation in vivo, with potential implications for liver regeneration and transplantation therapies.

## Significance Statement

The transcription factor ZNF143, which contains seven zinc finger domains, has been extensively studied in various cancers. However, its role and underlying mechanisms in liver regeneration remain largely unexplored. Here, we report the dynamic expression pattern of ZNF143 in a mouse liver regeneration model, suggesting its potential involvement in hepatocyte proliferation. Utilizing two cell lines, AML12 and Huh7, our study revealed that ZNF143 exerts opposing effects on cell proliferation and the cell cycle in normal hepatocytes versus hepatocellular carcinoma cells. Mechanistically, ZNF143 regulates these processes by suppressing c‐Myc expression. Future work focused on designing targeted therapies based on the regulatory model of ZNF143 could represent a significant strategy for addressing challenges in both liver regeneration and liver disease.

**Abbreviations**
CCK‐8: Cell Counting Kit‐8
CDC6: cell division cycle 6
CTCF: CCCTC‐binding factor
DAPI: 4′,6‐diamidino‐2‐phenylindole
EdU: 5‐ethynyl‐2′‐deoxyuridine
H3K9me3: histone H3 trimethylated at lysine 9
HCC: hepatocellular carcinoma
MDIG: mineral dust‐induced gene
MTT: 3‐(4,5‐dimethylthiazol‐2‐yl)‐2,5‐diphenyltetrazolium bromide
OE: overexpression
PHx: partial hepatectomy
qRT‐PCR: quantitative real‐time PCR
ZNF143: zinc finger protein 143

## 1 Introduction

The liver serves as the body's primary metabolic organ [1, 2] and possesses extraordinary regenerative capabilities [3]. Hepatocyte proliferation, growth, and differentiation during liver regeneration are crucial for maintaining liver function and homeostasis [4, 5]. However, the molecular mechanisms underlying liver regeneration involve intricate networks of transcription factors and signaling pathways [6], necessitating deeper investigation into regeneration‐associated genes and their mechanisms to advance therapeutic development.

ZNF143 is a well‐characterized transcriptional regulator primarily involved in cell cycle progression and DNA replication [7]. While its functional role varies across cellular contexts and disease states, ZNF143 generally promotes cell proliferation in most systems [8, 9]. A notable example is its oncogenic function in hepatocellular carcinoma (HCC), where ZNF143 activates transcription of the histone demethylase mineral dust‐induced gene (MDIG), subsequently upregulating CDC6 expression to drive tumor cell proliferation [10]. However, we have previously demonstrated that ZNF143 exhibits an opposing, growth‐suppressive function in normal hepatocytes, as evidenced by its ability to inhibit proliferation in rat BRL‐3A liver cells upon overexpression (OE) [11]. The proto‐oncogene c‐Myc, another key transcriptional regulator, plays pivotal roles in controlling gene expression programs and cell cycle checkpoint regulation [12]. Consistent with its fundamental role in cell cycle control, c‐Myc depletion invariably results in cell cycle arrest [13, 14]. While both ZNF143 and c‐Myc have been linked to cell proliferation and the cell cycle, it has been reported that ZNF143 primarily functions in the G2/M phase, whereas c‐Myc operates in the G0/G1 phase. Together, these observations indicate that both transcription factors are involved in cell cycle regulation, albeit at different phases [15, 16]. Nevertheless, the mechanism by which ZNF143 and c‐Myc cooperatively regulate the hepatocyte cell cycle remains poorly understood.

Intriguingly, ZNF143 activates MDIG to induce H3K9me3 demethylation at CDC6, thereby driving HCC proliferation [10]. Emerging evidence indicates that c‐Myc promotes liver regeneration by upregulating MDIG to mediate H3K9me3 demethylation at the OTX2 locus [17]. Therefore, ZNF143 and c‐Myc play distinct roles in HCC proliferation and liver regeneration through analogous regulatory mechanisms. To further investigate the regulatory interplay between ZNF143 and c‐Myc, recent findings have demonstrated that both factors promote the development and progression of liver cancers and glioblastoma, respectively, via their common target gene RIOX2 [10, 18, 19]. Moreover, it has been shown that the ZNF143–EIF4A–c‑Myc axis is critically involved in the regulation of lung metastasis in vivo [20]. Notably, recent studies demonstrate that ZNF143 depletion markedly reduces expression of c‐Myc target genes [21], suggesting potential functional crosstalk between these transcription factors in proliferation regulation. The aforementioned studies provide evidence that ZNF143 serves as an upstream transcription factor of c‑Myc and participates in related biological functions by modulating c‑Myc expression.

However, the cooperative mechanisms by which ZNF143 and c‐Myc coordinate hepatocyte proliferation remain elusive. Based on this, we first examine the expression of ZNF143 and c‐Myc during distinct phases of liver regeneration. We then employed two cell lines: normal mouse hepatocytes AML12 and human hepatoma cells Huh7 to: (1) determine their individual and combined effects on proliferation and cell cycle progression; (2) assess changes in cell cycle‐related gene expression; and (3) elucidate whether ZNF143 exerts dominant regulatory control over c‐Myc in this process. Our work offers important clues for further investigating how ZNF143 regulates hepatocyte proliferation to mediate liver regeneration processes in vivo.

## 2 Materials and Methods

### 2.1 Mouse Liver Regeneration Model

Eight‐week‐old male C57BL/6 mice were purchased from Beijing Vital River Laboratory Animal Technology Co. Ltd., Beijing, China. The 2/3 partial hepatectomy (PHx) was performed according to Mitchell and Willenbring [22]. Liver tissues were harvested at 0, 2, 36, 48, 72, and 168 h post‐surgery (18 mice in total, 6 time points, 3 mice per time point). All mice were housed in a light–dark cycle environment with free access to water and food. All animal experiments were conducted in accordance with procedures approved by the ethics committee at Henan Normal University, Henan, China, and conformed to the relevant regulatory standards. All animal studies were completed in the experimental animal center of Henan Normal University, China (License Number: HNSD‐2025‐07‐07, licensed by the Ministry of Science and Technology of China).

### 2.2 Cell Culture and Construction of Stable Transformed Cell Lines

Mouse hepatocyte AML12 cells (RRID: CRL‐2254, ATCC, Manassas, VA, USA) were cultured in Dulbecco's modified Eagle's medium (DMEM)/F‐12 medium (Hyclone) supplemented with 10% fetal bovine serum (FBS, Gibco, Grand Island, NY), 1x ITS‐G and 100 nM dexamethasone, HEK293T cells (RRID: CRL‐3216, ATCC, Manassas, VA, USA) were cultured using DMEM containing 10% FBS. Huh7 cells (RRID: SCSP‐526) were purchased from the Shanghai Institute of Life Sciences, Chinese Academy of Sciences. Cells were cultured in DMEM (Hyclone) supplemented with 10% FBS (Gibco, Grand Island, NY). All cell lines were incubated in a 5% CO_2_ humidified incubator and 1% penicillin–streptomycin was added to all culture media. All cell lines have been authenticated for the described experiments and have not been previously reported as misidentified or contaminated. All cell lines were examined for *Mycoplasma* contamination using the Myco‐Lumi Luminescent Detection Kit (C0298S, Beyotime, Shanghai), and none were found to be infected.

Plasmids containing the target gene were constructed using PCR amplification and restriction enzyme digestion. Viral particles were generated using HEK293T cells and then transfected into the AML12 cell line. Transfected cells were selected with puromycin to obtain stable cell lines expressing the target gene. Primers are listed in Table S1.

### 2.3 RNA Interference and Overexpression (OE)

Plasmids containing the target gene for transient OE were constructed using PCR amplification and restriction enzyme digestion methods, and interference fragments were synthesized by Sangon Biotech Co. Ltd. According to the instructions for Lipofectamine 2000 (Invitrogen), the oligomer‐Lipofectamine 2000 complex was prepared. The complex was added to the six‐well plate, and after incubation for 4–6 h, fresh medium was replaced. Huh7 cells were harvested 48 h later, and total RNA and protein were extracted. To examine cell proliferation/viability after transfection, 3 × 10^3^ cells per well were cultured in a 96‐well plate. ZNF143‐targeting interference sequence is 5′‐GGAAGAUAAUUGUACUAAATT‐3′. The primers for transient OE plasmids are listed in Table S2.

### 2.4 3‐(4,5‐Dimethylthiazol‐2‐yl)‐2,5‐diphenyltetrazolium Bromide (MTT) Assay

The MTT assay was performed using MTT purchased from Aladdin (Shanghai, China). Cells were seeded into 96‐well plates at a density of 3 × 10^3^ cells per well, and transfection was conducted after the cells fully adhered to the plate. After culturing for 48 h and 72 h, MTT solution (5 mg/mL) was added under light‐protected conditions, followed by incubation for 4 h under light protection. Following incubation, the supernatant was discarded, and 100 μL of DMSO was added to each well under light protection. The plate was shaken on a shaker until the blue crystals were completely dissolved. The absorbance at a wavelength of 490 nm was measured using a spectrophotometer.

### 2.5 Cell Counting Kit‐8 (CCK‐8) Assay

CCK‐8 reagent was purchased from Beijing Solarbio Biotechnology Co. Ltd. Cells were seeded into 96‐well plates at a density of 3 × 10^3^ cells per well, and transfection was conducted after the cells fully adhered to the plate. After culturing for 48 and 72 h, the cell culture solution was discarded first, and 1 mL of CCK‐8 working solution (CCK‐8: complete medium = 1:10) was added to each well. The absorbance at a wavelength of 450 nm was measured using a spectrophotometer.

### 2.6 5‐Ethynyl‐2′‐deoxyuridine (EdU) Assay

Cells were seeded into 96‐well plates at a density of 3 × 10^3^ cells per well, and transfection was conducted after the cells fully adhered to the plate. The cells were stained according to the instructions of the EdU kit (RiboBio, Guangzhou, China). Fluorescence microscopy (Zeiss) with excitation/emission filters of Ex/Em = 546/565 nm was used to analyze the labeled DNA in the samples, while the DNA replication status of the cell nuclei was detected using Ex/Em = 360/460 nm.

### 2.7 RNA Sample Preparation and qRT‐PCR

Total RNA was extracted using the TRIzol Reagent (QIAGEN) and subsequently reverse‐transcribed into cDNA using the GoScript Reverse Transcription System (Promega). Quantitative real‐time PCR (qRT‐PCR) was performed on the LightCycler 96 instrument (Roche) using GoTaq qPCR Master Mix (Promega), following the manufacturer's instructions. GAPDH was used as the internal reference. Primers are listed in Table S3.

### 2.8 Western Blot

Cell lysis buffer was purchased from Beyotime Biotechnology Co. Ltd. Protein samples were prepared using the lysis buffer; electrophoresis and wet transfer (SDS‐PAGE to PVDF membrane) were performed; primary antibodies were incubated overnight, and secondary antibodies were incubated at room temperature for 1.5 h; bands were visualized using ECL chemiluminescence reagent and Odyssey XF (LI‐COR Biosciences), and protein content was analyzed using ImageJ software. The antibodies used in this study are listed in Table S4.

### 2.9 Statistical Analysis

Data processing was performed using GraphPad Prism 6.0 software. The data are presented as the mean ± standard error of the mean (SEM) [23]. Differences between groups were analyzed using an independent *t*‐test. A *p* value of less than 0.05 was considered statistically significant.

## 3 Results

### 3.1 Dynamic Expression of ZNF143 and c‐Myc During Distinct Phases of Liver Regeneration

In a murine 2/3 PHx model, liver tissues were collected at six time points: 0, 2, 36, 48, 72, and 168 h, with triplicate biological replicates at each interval. Initial assessment of the liver‐to‐body weight ratio confirmed successful liver regeneration, demonstrating progressive weight restoration over time (Figure 1A). Subsequently, we characterized the dynamic expression profiles of ZNF143 and c‐Myc at both mRNA and protein levels during liver regeneration. The results indicated that both ZNF143 and c‐Myc exhibited dynamic changes during the initiation and proliferation phases of regeneration, with opposing trends. Specifically, at 36 h, c‐Myc was significantly upregulated while ZNF143 was significantly downregulated (Figure 1B–D). Meanwhile, we observed that during the 48–168‐h period following PHx, the expression trends of ZNF143 and c‑Myc (at both mRNA and protein levels) remained opposite. Notably, at 48 h post‑PHx, ZNF143 mRNA was barely detectable (Figure 1B), whereas its protein level was relatively prominent. We therefore speculate that, at this time point, the protein half‑life of ZNF143 is considerably longer than its mRNA half‑life, likely as a result of the in vivo transcriptional and translational context. By contrast, c‑Myc mRNA was relatively abundant at 48 h, yet its protein was almost undetectable. We attribute this to the inherently short half‑life of c‑Myc protein, and possibly also to proteasome‑mediated rapid degradation in vivo. The expression patterns of ZNF143 and c‐Myc during the proliferation phase, particularly their impact on hepatocyte proliferation, as well as how they jointly regulate liver regeneration, warrant further investigation.

**Figure 1:**
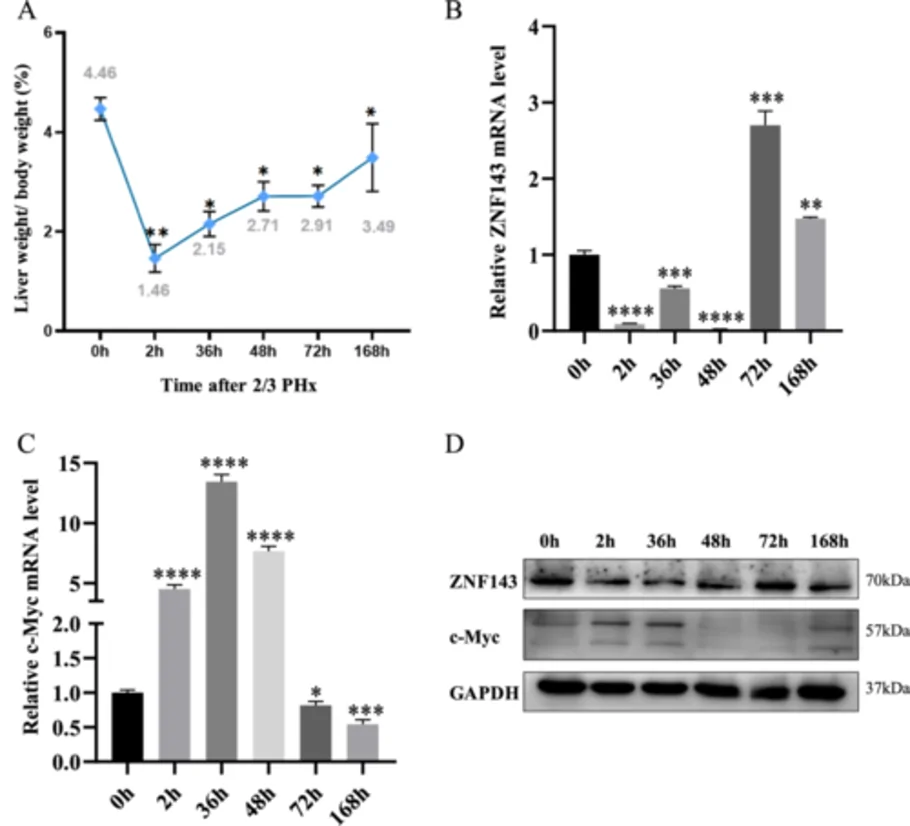
Detection of ZNF143 and c‐Myc expression during liver regeneration. (A) Liver‐to‐body weight ratio at different time points after 2/3 PHx in mice (3 mice per group). (B) Changes in ZNF143 mRNA levels. (C) Changes in c‐Myc mRNA levels. (D) Changes in ZNF143 and c‐Myc protein levels. GAPDH was used as the loading control. All data are presented as mean ± SEM, *n* = 3 (three biological replicates), statistical significance was determined by a two‐sided Student's *t* test, with the control group (set as the reference baseline) used for pairwise comparisons. *p < 0.05, **p < 0.01, ***p < 0.001, ****p < 0.0001.

### 3.2 ZNF143 Inhibits the Proliferation of Normal Hepatocytes In Vitro

To evaluate the role of ZNF143 in hepatocyte proliferation, we first constructed a stable ZNF143‐knockdown AML12 cell line. The qRT‐PCR analysis confirmed an 84.5% reduction in ZNF143 mRNA expression following shRNA‐mediated ZNF143 knockdown (Figure 2A). Subsequently, cell viability and proliferation were assessed using CCK‐8, MTT, and EdU assays, which revealed that ZNF143 knockdown significantly promoted both cell viability and proliferation in AML12 cells (Figure 2B–E). Finally, to determine the impact of ZNF143 on the expression of genes related to the cell cycle and cell proliferation, we found that after ZNF143 knockdown, CCNA2, CCND1, CCNB1, CCNE1, c‐Jun, and PCNA were significantly upregulated at both mRNA and protein levels in AML12 cells (Figure 2F–H). These findings collectively indicate that knocking down ZNF143 promotes the proliferation of AML12 cells, suggesting that ZNF143 knockdown accelerates normal hepatocyte proliferation.

**Figure 2:**
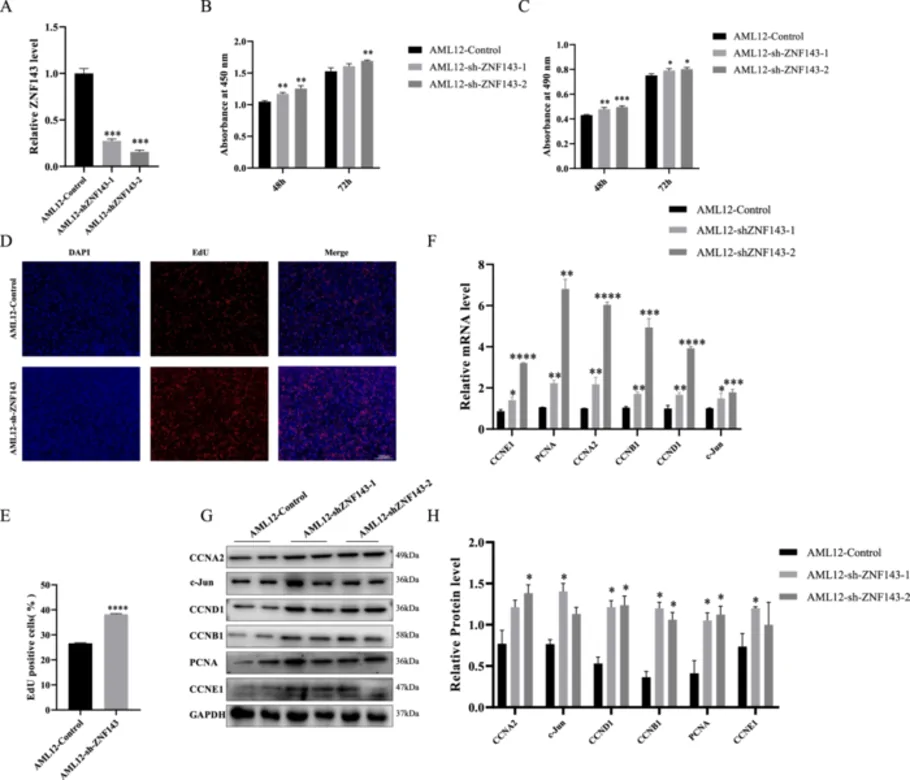
The effects of ZNF143 knockdown on the expression of cell proliferation and cell cycle‐related genes in AML12 cells. (A) qRT‐PCR analysis of ZNF143 mRNA levels. (B) CCK‐8 assay evaluating AML12 cell viability at 48 and 72 h after ZNF143 knockdown. (C) MTT assay evaluating AML12 cell viability at 48 and 72 h after ZNF143 knockdown. (D) EdU fluorescence imaging; 4′,6‐diamidino‐2‐phenylindole (DAPI) was used for nuclear staining, and EdU incorporation was used to label proliferating cells. (E) Analysis of EdU‐positive cell rates. (F) qRT‐PCR analysis of the mRNA levels of proliferation and cell cycle‐related genes. (G) Western blot analysis of the protein levels of proliferation and cell cycle‐related genes; GAPDH was used as the loading control. (H) Grayscale analysis of the bands in (G). All data are presented as mean ± SEM, *n* = 3 (three biological replicates), statistical significance was determined by a two‐sided Student's *t* test, with the control group (set as the reference baseline) used for pairwise comparisons. *p < 0.05, **p < 0.01, ***p < 0.001, ****p < 0.0001.

Subsequently, by overexpressing ZNF143 in AML12 cells, the results showed a significant decrease in cell viability and proliferation (Figure S1A–E). Similarly, ZNF143 OE significantly suppressed the expression of the aforementioned cell cycle and cell proliferation‐related genes (Figure S1F–H). Thus, these experiments demonstrate that OE of ZNF143 inhibits the proliferation of AML12 cells, indicating that ZNF143 OE suppresses normal hepatocyte proliferation. These results collectively suggest that ZNF143 is a suppressor of cell proliferation in normal hepatocytes in vitro.

### 3.3 ZNF143 Promotes the Proliferation of HCC Cells In Vitro

The above research indicates that ZNF143 inhibits the proliferation of normal hepatocyte cell line AML12. We next sought to examine the impact of ZNF143 on the proliferation of the HCC cell line Huh7. We synthesized siRNA targeting human ZNF143 to investigate the function of ZNF143 in Huh7 cells. First, qRT‐PCR analysis confirmed efficient knockdown of ZNF143 (58% reduction) in Huh7 cells following siRNA transfection (Figure 3A). Subsequently, the CCK‐8, MTT, and EdU assays revealed that ZNF143 knockdown significantly reduced the viability and proliferation of Huh7 cells (Figure 3B–E). Finally, qRT‐PCR and western blot analysis demonstrated that ZNF143 knockdown significantly increased the mRNA and protein levels of genes associated with cell proliferation and cell cycle in Huh7 cells (Figure 3F–H). To further investigate the role of ZNF143 in Huh7 cells, we generated a ZNF143‐overexpressing cell model using the pcDNA3.1‐ZNF143 plasmid. The results revealed that ZNF143 OE significantly enhanced Huh7 cell viability and proliferation (Figure S2A–H). Overall, these results show that ZNF143 promotes Huh7 cell proliferation.

**Figure 3:**
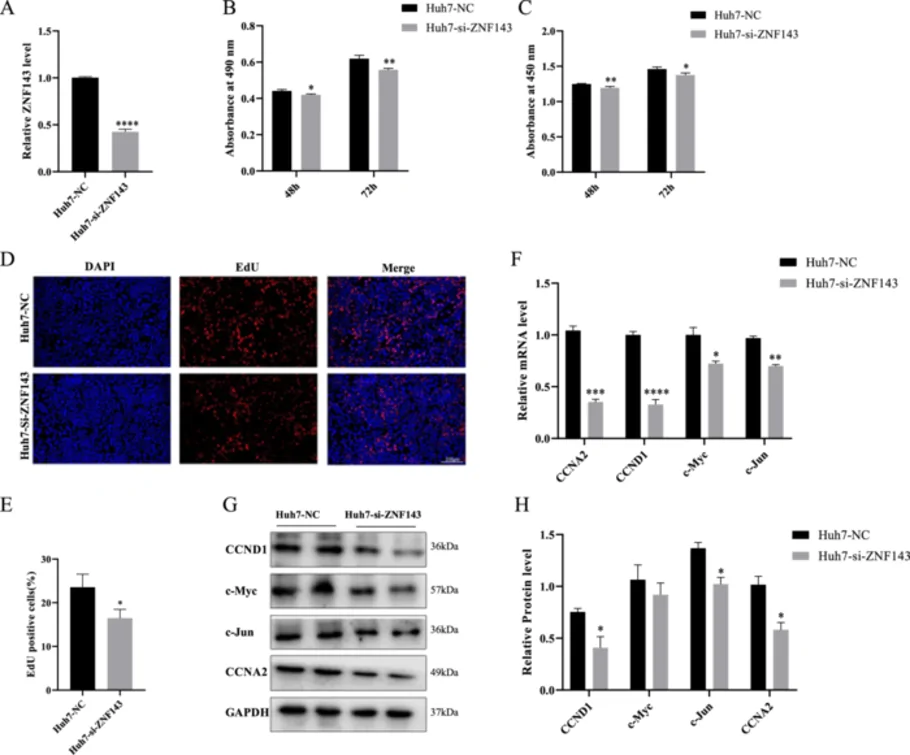
The effects of ZNF143 knockdown on the expression of cell proliferation and cycle‐related genes in Huh7 cells. (A) qRT‐PCR analysis of ZNF143 mRNA levels. (B) CCK‐8 assay evaluating Huh7 cell viability at 48 and 72 h after ZNF143 knockdown. (C) MTT assay evaluating Huh7 cell viability at 48 and 72 h after ZNF143 knockdown. (D) EdU fluorescence imaging; DAPI was used for nuclear staining, and EdU incorporation was used to label proliferating cells. (E) Analysis of EdU‐positive cell rates. (F) qRT‐PCR analysis of the mRNA levels of proliferation and cycle‐related genes. (G) Western blot analysis of the protein levels of proliferation and cycle‐related genes. GAPDH was used as the loading control. (H) Grayscale analysis of the bands in (G). All data are presented as mean ± SEM, *n* = 3 (three biological replicates), statistical significance was determined by a two‐sided Student's *t* test, with the control group (set as the reference baseline) used for pairwise comparisons. *p < 0.05, **p < 0.01, ***p < 0.001, ****p < 0.0001.

### 3.4 ZNF143 Synergizes With c‐Myc to Inhibit Hepatocyte Proliferation

Recent studies have shown that ZNF143 depletion leads to significant downregulation of c‐Myc target genes [21], indicating that ZNF143 may regulate hepatocyte proliferation by controlling the expression of c‐Myc target genes. During the proliferative phase of liver regeneration, we observed a downregulation of ZNF143 expression, particularly at 36 h, while c‐Myc expression was upregulated (compared to 0 h) (Figure 1B–D). In normal hepatocytes, studies have found that upregulation of ZNF143 expression inhibits proliferation, while upregulation of c‐Myc expression promotes proliferation [17]. Therefore, it is speculated that ZNF143 (downregulated) synergizes with c‐Myc (upregulated) to jointly promote hepatocyte proliferation. To investigate the relationship between these two factors in the regulation of cell proliferation, we constructed a stable c‐Myc‐knockdown AML12 cell line, and the experiments showed that c‐Myc knockdown inhibited the viability and proliferation of AML12 cells (Figure S3A–E), consistent with previous reports [17]. Furthermore, we found that, in AML12 cells, OE of ZNF143 significantly inhibited the expression of c‐Myc, while knockdown of ZNF143 significantly promoted the expression of c‐Myc (Figure 4A–F). Therefore, in AML12 cells, c‐Myc is a regulatory factor that promotes hepatocyte proliferation, while ZNF143 attenuates the potential for hepatocyte proliferation by inhibiting the expression of c‐Myc.

**Figure 4:**
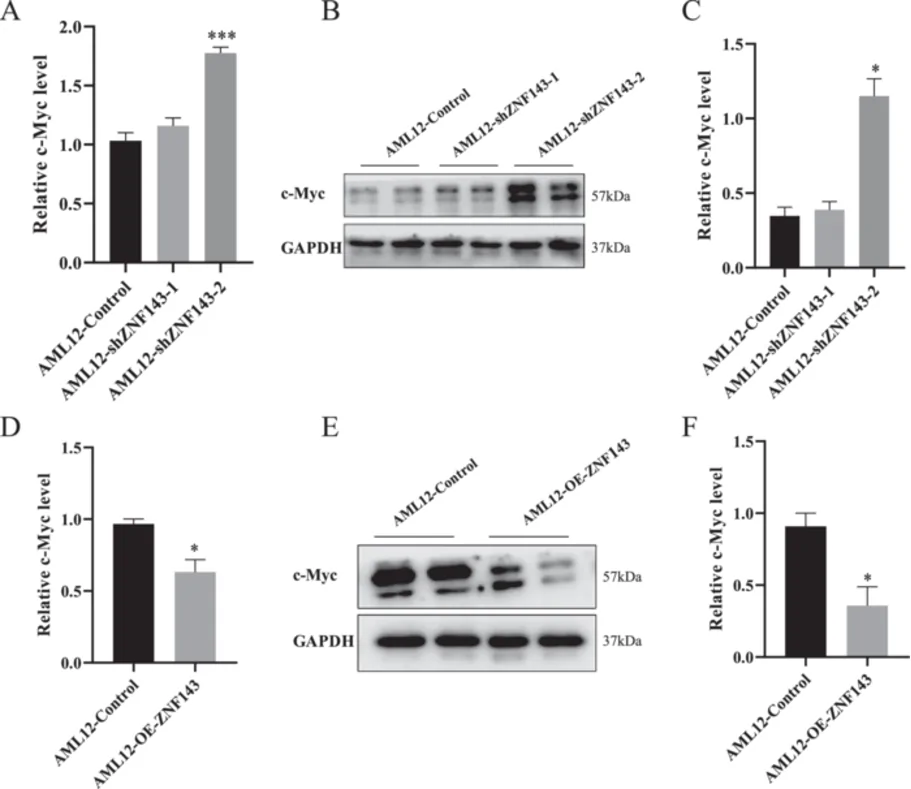
ZNF143 regulates the expression of c‐Myc in AML12 cells. (A) qRT‐PCR analysis of c‐Myc mRNA levels in AML12 cells upon ZNF143 knockdown. (B) Western blot analysis of c‐Myc protein levels in AML12 cells upon ZNF143 knockdown; GAPDH was used as the loading control. (C) Grayscale analysis of the bands in (B). (D) qRT‐PCR analysis of c‐Myc mRNA levels in AML12 cells upon ZNF143 OE; GAPDH was used as the loading control. (E) Western blot analysis of c‐Myc protein levels in AML12 cells upon ZNF143 OE; GAPDH was used as the loading control. (F) Grayscale analysis of the bands in (E) All data are presented as mean ± SEM, *n* = 3 (three biological replicates), statistical significance was determined by a two‐sided Student's *t* test, with the control group (set as the reference baseline) used for pairwise comparisons. *p < 0.05, ***p < 0.001.

To further investigate the relationship between ZNF143 and c‐Myc, we co‐overexpressed ZNF143 and c‐Myc in AML12 cells. The results showed that OE of c‐Myc promoted the proliferation of AML12 cells, whereas OE of ZNF143 inhibited cell proliferation. When both c‐Myc and ZNF143 were co‐overexpressed, although c‐Myc partially counteracted the inhibitory effect of ZNF143, the proliferation of hepatocytes remained suppressed (Figure 5A–H). Simultaneously, when ZNF143 and c‐Myc were co‐overexpressed, the expression of cell cycle and proliferation‐related genes remained significantly suppressed (Figure 5I,J). Taken together, these findings suggest that co‐OE of ZNF143 and c‐Myc in AML12 cells inhibits cell proliferation. ZNF143 maintains the identity of normal hepatocytes by inhibiting the excessive proliferation of normal hepatocytes through the suppression of c‐Myc expression.

**Figure 5:**
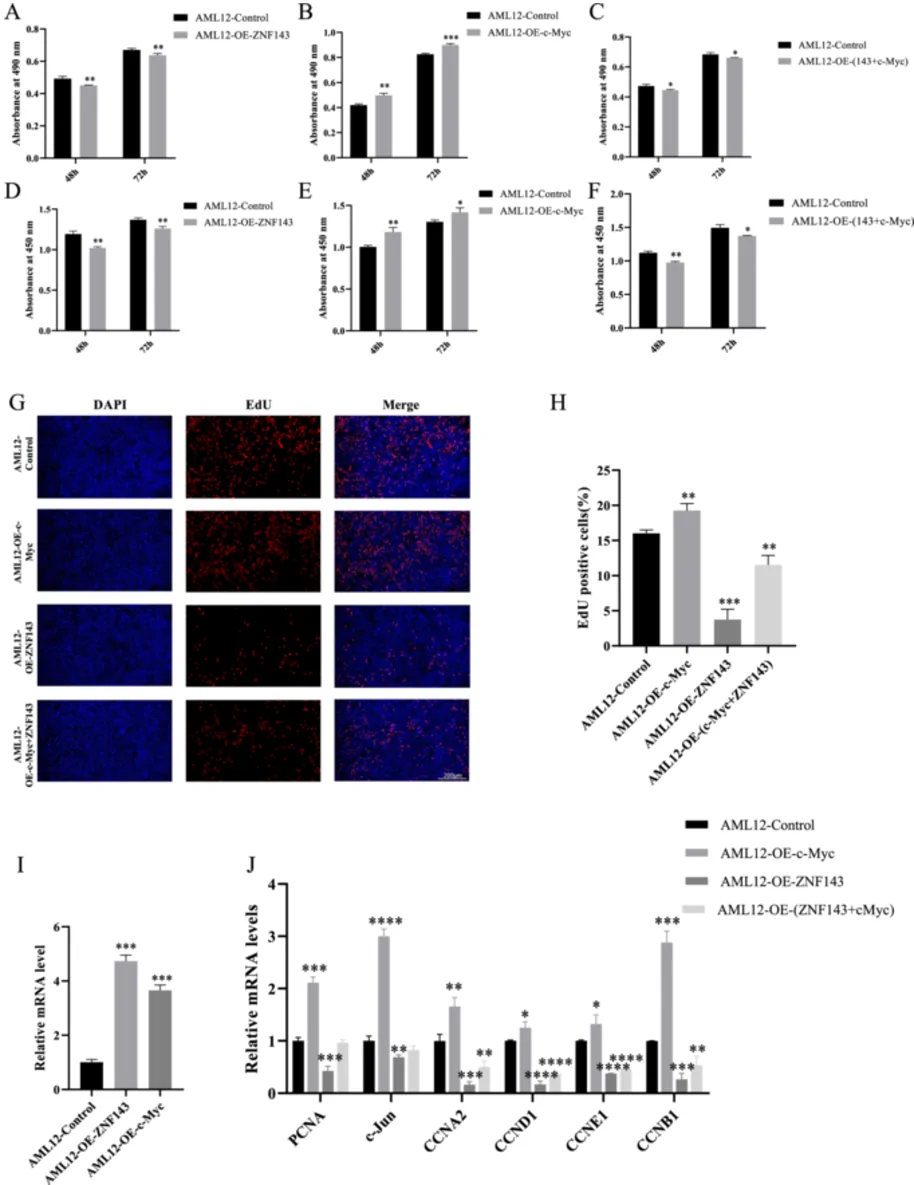
The effects of OE of ZNF143, c‐Myc, and their co‐OE on cell viability and proliferation in AML12 cells. (A) MTT assay evaluating AML12 cell viability at 48 and 72 h after ZNF143 OE. (B) MTT assay evaluating AML12 cell viability at 48 and 72 h after c‐Myc OE. (C) MTT assay evaluating AML12 cell viability at 48 and 72 h after co‐OE of ZNF143 and c‐Myc. (D) CCK‐8 assay evaluating AML12 cell viability at 48 and 72 h after ZNF143 OE. (E) CCK‐8 assay evaluating AML12 cell viability at 48 and 72 h after c‐Myc OE. (F) CCK‐8 assay evaluating AML12 cell viability at 48 and 72 h after co‐OE of ZNF143 and c‐Myc. (G) EdU fluorescence imaging; DAPI was used for nuclear staining, and EdU incorporation was used to label proliferating cells. (H) Analysis of EdU‐positive cell rates. (I) qRT‐PCR analysis of ZNF143 and c‐Myc mRNA levels. (J) qRT‐PCR analysis of the mRNA levels of cell proliferation and cell cycle‐related genes. All data are presented as mean ± SEM, *n* = 3 (three biological replicates), statistical significance was determined by a two‐sided Student's *t* test, with the control group (set as the reference baseline) used for pairwise comparisons. *p < 0.05, **p < 0.01, ***p < 0.001, ****p < 0.0001.

## 4 Discussion

In recent years, studies have shown that ZNF143 is highly expressed in various solid tumors, and its target genes are mostly related to cell proliferation. For example, ZNF143 can promote the expression of CDC6 by activating the transcription of MDIG, thereby accelerating the progression of the cell cycle in HCC cells [10]. Although numerous experimental studies have demonstrated that ZNF143 acts as an activator in cell proliferation, recent research has found that ZNF143 also has inhibitory effects. In prostate cancer PC3 cells, either downregulation of ZNF143 or forced OE of ZNF143 decreased cell proliferation [9]. Our recent research revealed that OE of ZNF143 in BRL‐3A cells also inhibits cell proliferation [11]. In this work, we found that ZNF143 exhibits dynamic changes during the initiation and proliferation phases of regeneration. ZNF143 is downregulated at the initiation stage of liver regeneration and upregulated during the proliferation phase, revealing that ZNF143 may have different effects on regeneration at different stages. Meanwhile, we found that ZNF143 uniformly inhibited the proliferation of normal hepatocytes (AML12 cells). However, in Huh7 HCC cells, ZNF143 promotes cell proliferation. These results indicated that the role of ZNF143 in cell proliferation is complex and diverse, potentially promoting or inhibiting proliferation depending on the context. This dual role reflects the multifunctionality of ZNF143 in different biological contexts and provides important clues for exploring its potential therapeutic targets in diseases.

The transcription factor c‐Myc plays a pivotal role in cell proliferation, differentiation, and metabolic regulation [24]. Previous studies have demonstrated that c‐Myc activation enhances liver regeneration [25]. Notably, recent research revealed that c‐Myc upregulates MDIG expression, leading to reduced H3K9me3 levels at the OTX2 locus, thereby promoting liver regeneration [17]. Intriguingly, ZNF143 similarly upregulates MDIG, which decreases H3K9me3 methylation at the CDC6 gene, subsequently upregulating CDC6 and driving the proliferation of HCC [10]. Furthermore, ZNF143 depletion has been shown to significantly downregulate c‐Myc target genes [21], suggesting potential direct or indirect crosstalk between ZNF143 and c‐Myc. Moreover, relevant studies have shown that the expression of c‐Myc is regulated by ZNF143 [18, 19, 20]. In this study, we observed dynamic changes in c‐Myc expression during the initiation and proliferation phases of liver regeneration in a mouse 2/3 PHx model. While c‐Myc was markedly upregulated during the proliferative phase, its expression pattern contrasted with that of ZNF143, implying a functional interplay between these two factors in liver regeneration. In fact, we co‐overexpressed ZNF143 and c‐Myc in AML12 cells and found that the effect on cell proliferation was consistent with that of overexpressing ZNF143 alone. Combined with the aforementioned literature reports, we conclude that the expression of c‐Myc is regulated by ZNF143, thereby achieving precise regulation of cell proliferation. Although we investigated the combined effects of ZNF143 and c‐Myc on hepatocyte proliferation through in vitro hepatocyte cell lines. Whether ZNF143 maintains the smooth progression of liver regeneration by regulating c‐Myc still requires more in‐depth research. However, our results suggest that c‐Myc and ZNF143 play different roles in regulating cell proliferation and may jointly regulate hepatocyte proliferation during liver regeneration to prevent excessive regeneration and subsequent carcinogenesis.

## 5 Conclusion

As a transcription factor, ZNF143 typically mediates the formation of loops together with the chromatin organizer protein CCCTC‐binding factor (CTCF) [26, 27]. However, recent reports indicate that the primary role of ZNF143 is to regulate its target genes, such as nuclear‐encoded mitochondrial genes, independent of loops and CTCF [16, 28, 29]. In the present study, we found that ZNF143 suppresses cell proliferation in normal hepatocytes (AML12) but enhances it in hepatoma cells (Huh7), whereas c‑Myc promotes proliferation in both cell lines. c‑Myc expression is negatively regulated by ZNF143. Moreover, in AML12 cells, the proliferative effect of simultaneous OE of ZNF143 and c‑Myc is similar to that of ZNF143 alone (Figure 6). Thus, we propose that ZNF143, as a pivotal transcription factor governing cell proliferation, exerts a predominant role in the regulation of hepatocyte proliferation. Although this study investigated the effects of coordinated expression of ZNF143 and c‐Myc on hepatocyte proliferation, the specific molecular mechanisms remain unclear. In the future, we will further explore the molecular mechanisms by which ZNF143 inhibits the proliferation of normal hepatocytes. Additionally, we will study how ZNF143 and c‐Myc regulate the changes in H3K9me3 of target genes to exert their respective functions. In conclusion, our research indicates that ZNF143 plays different roles in HCC and normal hepatocytes and collaborates with c‐Myc to regulate hepatocyte proliferation. It can be considered a potential therapeutic target for liver diseases.

**Figure 6:**
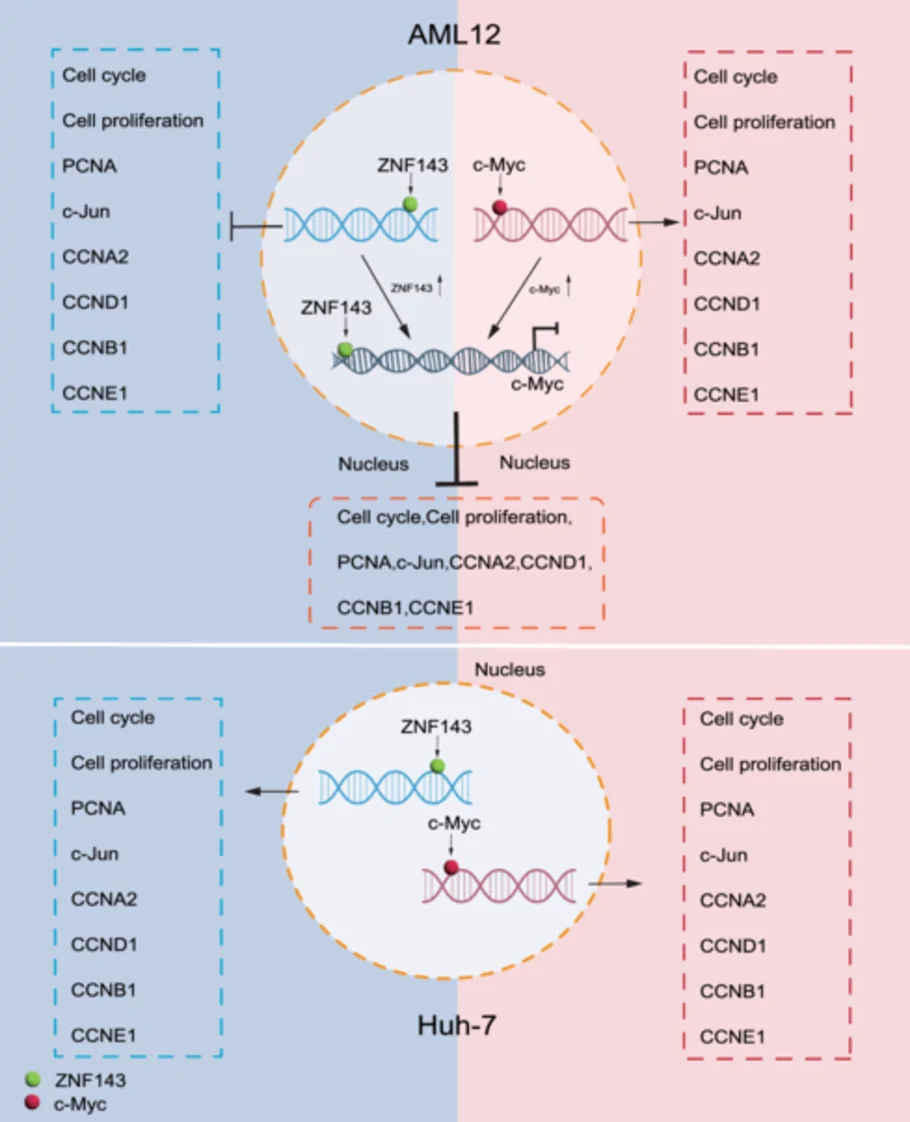
A proposed model illustrating the role of ZNF143 and c‑Myc in the proliferation of normal (AML12) and hepatoma (Huh7) cell lines. The left and right panels represent AML12 and Huh7 cells, respectively. In AML12 cells, ZNF143 inhibits c‐Myc expression and suppresses cell cycle progression and proliferation. In Huh7 cells, ZNF143 promotes cell cycle progression and proliferation. Green circles represent ZNF143; red circles represent c‐Myc. Arrows indicate promotion, and blunt‐ended lines indicate inhibition. The figure was created with the assistance of Adobe Illustrator (24.0.3).

## Author Contributions

Bingyu Ye conceived and designed the project. Caifang Sun and Meijuan Yue carried out the experiments. Yuxiang Chen, Jiaying Zhang, Yunyun Mai, and Yuhang Zhang carried out the data analysis. Caifang Sun and Bingyu Ye contributed to results interpretation and wrote the manuscript. All authors read and approved the final manuscript.

## Ethics Statement

All animal experiments were conducted in accordance with procedures approved by the Ethics Committee at Henan Normal University, Henan, China, and conformed to the relevant regulatory standards. All animal studies were completed in the experimental animal center of Henan Normal University, China (License Number: HNSD‐2025‐07‐07, licensed by the Ministry of Science and Technology of China).

## Consent

The authors have nothing to report.

## Conflicts of Interest

The authors declare no conflicts of interest.

## Supporting information


Supporting File

## Data Availability

The authors have nothing to report.
