# Supplementary material for: Zinc Finger Protein 143 Bidirectionally Regulates the Proliferation of Hepatocytes and Hepatocellular Carcinoma Cells via c‐Myc
Source: Cell Biochem Funct. 2026 Oct 7;44(10):e70320. doi: 10.1002/cbf.70320 (PMC13643501; doi:10.1002/cbf.70320)
Supplement: Supplementary file 1 [file CBF-44-e70320-s001.docx]

Table S1 The primer sequences used for stable expression plasmid construction

| Oligo name | Primer sequences（5’→3’） |
| --- | --- |
| OE-MYC-F | CGCGGATCCGCCACCATGCCCCTCAACGTGAACTTCACC |
| OE-MYC-R | CCGCTCGAGTTATGCACCAGAGTTTCGAAG |
| OE-ZNF143-F | CGGGGTACCGCCACCATGTTGTTAGCACAAATAAATCGG |
| OE-ZNF143-R | CCGCTCGAGTTAATCATCCAACCCTGGCGTC |
| shRNA-ZNF143-F-1 | CCGGGctactcgggtaactcctaaaCTCGAGtttaggagtt  acccgagtagCTTTTTG |
| shRNA-ZNF143-R-1 | AATTCAAAAAGctactcgggtaactcctaaaCTCGAGttt  aggagttacccgagtagC |
| shRNA-ZNF143-F-2 | CCGGAgaggacattgtatctacacaagtaCTCGAGtactt  gtgtagatacaatgtcctcTTTTTTG |
| shRNA-ZNF143-R-2 | AATTCAAAAAAgaggacattgtatctacacaagtaCTCGAG  tacttgtgtagatacaatgtcctcT |
| shRNA-c-Myc-F-1 | CCGGGCTCTCATCATCTGCAACAAACTCGAGTTTGTTGCAG  ATGATGAGAGCTTTTTG |
| shRNA-c-Myc-R-1 | AATTCAAAAAGCTCTCATCATCTGCAACAAACTCGAGTTTG  TTGCAGATGATGAGAGC |
| shRNA-c-Myc-F-2 | CCGGTGCCCACTATTGAGAGAGTATCTCGAGATACTCTCTCA  ATAGTGGGCATTTTTG |
| shRNA-c-Myc-R-2 | AATTCAAAAATGCCCACTATTGAGAGAGTATCTCGAGATACT  CTCTCAATAGTGGGCA |

Table S2 The primer sequences used for transient plasmids construction

| Oligo Name | Primer sequences（5’→3’） |
| --- | --- |
| OE-MYC-F | CGCGGATCCGCCACCATGGATTACAAGGATGACGACGATAAGcccctcaacgtgaactt |
| OE-MYC-R | CCGCTCGAGTTATGCACCAGAGTTTCGAAG |
| OE-ZNF143-F | CGGGGTACCGCCACCATGTACCCATACGACGTCCCAGACTACGCTttgttagcacaaataaa |
| OE-ZNF143-R | CCGCTCGAGTTAATCATCCAACCCTGGCGTC |

Table S3 The qRT-PCR primers used in this study

| Genes | Primer Sequences（5’→3’） |
| --- | --- |
| Mouse ZNF143-FP | ATGTTACCCTGTGCCTGACG |
| Mouse ZNF143-RP | CGCAAACTGTCCCCTGTACT |
| Mouse GAPDH-FP | AGGTCGGTGTGAACGGATTTG |
| Mouse GAPDH-RP | TGTAGACCATGTAGTTGAGGTCA |
| Mouse PCNA-FP | GAACCTCACCAGCATGTCCA |
| Mouse PCNA-RP | ATTCACCCGACGGCATCTT T |
| Mouse c-Myc-FP | ATGTTGCGGTCGCTACGTC |
| Mouse c-Myc-RP | AGAAGTTGCCACCGCCG |
| Mouse c-Jun-FP | CGATGCCCTCAACGCC |
| Mouse c-Jun-RP | CTTAGGGTTACTGTAGCCGTAGG |
| Mouse CCNA2-FP | TCTGGGATTAAAGGCGCCAC |
| Mouse CCNA2-RP | CCGACGGGAATCTTACAGGG |
| Mouse CCNB1-FP | ACAACGGTGAATGGACACCA |
| Mouse CCNB1-RP | GTACAGTTCAGCTGTGCCA |
| Mouse CCND1-FP | AAAATGCCAGAGGCGGATGA |
| Mouse CCND1-RP | CAGGGCCTTGACCGGG |
| Mouse CCNE1-FP | GCTTCGGGTCTGAGTTCCAA |
| Mouse CCNE1-RP | GTCTTGCAAAAACACGGCCA |
| Human GAPDH-F | GTCTCCTCTGACTTCAACAGCG |
| Human GAPDH-R | ACCACCCTGTTGCTGTAGCCAA |
| Human PCNA-F | GCGTGAACCTCACCAGTATG |
| Human PCNA-R | ACGTGCAAATTCACCAGAAGG |
| Human CCNA2-F | CGCTGGCGGTACTGAAGTC |
| Human CCNA2-R | GAGGAACGGTGACATGCTCAT |
| Human CCND1-F | GCTGCGAAGTGGAAACCATC |
| Human CCND1-R | CCTCCTTCTGCACACATTTGAA |
| Human c-MYC-F | GCAATGCGTTGCTGGGTTATTT |
| Human c-MYC-R | AGAGTCCCAGGGAGAGTGGA |
| Human c-JUN–F | TGACTGCAAAGATGGAAACG |
| Human c-JUN-R | CAGGGTCATGCTCTGTTTCA |

| Antibody name | Manufacturer | Dilution ratio |
| --- | --- | --- |
| GAPDH | Affinity | 1：5000 |
| ZNF143 | Immunoway | 1：1000 |
| CCNA2 | Immunoway | 1：1000 |
| CCNB1 | Immunoway | 1：1000 |
| CCND1 | Immunoway | 1：1000 |
| CCNE1 | Immunoway | 1：1000 |
| PCNA | Affinity | 1：1000 |
| c-Myc | Affinity | 1：1000 |
| c-Jun | Affinity | 1：1000 |

Table S4 The antibodies used in this study

**Figure S1** The effects of ZNF143 OE on the expression of cell proliferation and cell cycle-related genes in AML12 cells

A. qRT-PCR analysis of ZNF143 mRNA levels; B. CCK-8 assay evaluating AML12 cell viability at 48h and 72h after ZNF143 OE; C. MTT assay evaluating AML12 cell viability at 48h and 72h after ZNF143 OE; D. EdU fluorescence imaging; E. Analysis of EdU-positive cell rates; F. qRT-PCR analysis of the mRNA levels of cell proliferation and cell cycle-related genes; G. Western blot analysis of the protein levels of cell proliferation and cell cycle-related genes; H Western blot density analysis. All data are presented as mean ± SEM, n=3 (three biological replicates), statistical significance was determined by a two-sided Student's *t* test, with the control group (set as the reference baseline) used for pairwise comparisons. *p < 0.05, **p < 0.01, ***p < 0.001, ****p < 0.0001.

**Figure S2** The effects of ZNF143 OE on the expression of cell proliferation and cell cycle-related genes in Huh7 cells

A. qRT-PCR analysis of ZNF143 mRNA levels; B. CCK-8 assay evaluating Huh7 cell viability at 48h and 72h after ZNF143 OE; C. MTT assay evaluating Huh7 cell viability at 48h and 72h after ZNF143 OE; D. EdU fluorescence imaging; E. Analysis of EdU-positive cell rates; F. qRT-PCR analysis of the mRNA levels of cell proliferation and cell cycle-related genes; G. Western blot analysis of the protein levels of cell proliferation and cell cycle-related genes; H. Western blot density analysis. All data are presented as mean ± SEM, n=3 (three biological replicates), statistical significance was determined by a two-sided Student's *t* test, with the control group (set as the reference baseline) used for pairwise comparisons. *p < 0.05, **p < 0.01, ***p < 0.001, ****p < 0.0001.

**Figure S3** The effects of c-Myc knockdown on proliferation and viability of AML12 cells

A. qRT-PCR analysis of c-Myc mRNA levels; B. CCK-8 assay evaluating AML12 cell viability at 48h and 72h after c-Myc knockdown; C. MTT assay evaluating AML12 cell viability at 48h and 72h after c-Myc knockdown; D. EdU fluorescence images; E Analysis of EdU-positive cell rates. All data are presented as mean ± SEM, n=3 (three biological replicates), statistical significance was determined by a two-sided Student's *t* test, with the control group (set as the reference baseline) used for pairwise comparisons. *p < 0.05, **p < 0.01, ***p < 0.001, ****p < 0.0001.
